# Prenatal Air Pollution Exposures, DNA Methyl Transferase Genotypes, and Associations with Newborn LINE1 and Alu Methylation and Childhood Blood Pressure and Carotid Intima-Media Thickness in the Children’s Health Study

**DOI:** 10.1289/EHP181

**Published:** 2016-05-24

**Authors:** Carrie V. Breton, Jin Yao, Josh Millstein, Lu Gao, Kimberly D. Siegmund, Wendy Mack, Lora Whitfield-Maxwell, Fred Lurmann, Howard Hodis, Ed Avol, Frank D. Gilliland

**Affiliations:** 1 Department of Preventive Medicine, and; 2 Atherosclerosis Research Unit, University of Southern California, Los Angeles, California, USA; 3 Sonoma Technology, Inc., Petaluma, California, USA

## Abstract

**Background:**

Although exposure to ambient air pollutants increases cardiovascular disease risk in adults little is known about the effects of prenatal exposure. Genetic variation and epigenetic alterations are two mechanisms that may influence the effects of early-life exposures on cardiovascular phenotypes.

**Objectives:**

We investigated whether genetic and epigenetic variation modify associations between prenatal air pollution on markers of cardiovascular risk in childhood.

**Methods:**

We used linear regression analysis to investigate the associations between prenatal pollutants (PM2.5, PM10, NO2, O3), long interspersed nuclear elements (LINE1) and AluYb8 DNA methylation levels measured in newborn blood spot tests, and carotid intima-media thickness (CIMT) and blood pressure (BP) in 459 participants as part of the Children’s Health Study. Interaction terms were also included to test for effect modification of these associations by genetic variation in methylation reprogramming genes.

**Results:**

Prenatal exposure to NO2 in the third trimester of pregnancy was associated with higher systolic BP in 11-year-old children. Prenatal exposure to multiple air pollutants in the first trimester was associated with lower DNA methylation in LINE1, whereas later exposure to O3 was associated with higher LINE1 methylation levels in newborn blood spots. The magnitude of associations with prenatal air pollution varied according to genotype for 11 SNPs within DNA methyltransferase 1 (DNMT1), DNA methyltransferase 3 Beta (DNMT3B), Tet methylcytosine dioxygenase 2 (TET2), and Thymine DNA glycosylase (TDG) genes. Although first-trimester O3 exposure was not associated with CIMT and systolic BP overall, associations within strata of DNMT1 or DNMT3B were observed, and the magnitude and the direction of these associations depended on DNMT1 genotypes.

**Conclusions:**

Genetic and epigenetic variation in DNA methylation reprogramming genes and in LINE1 retrotransposons may play important roles in downstream cardiovascular consequences of prenatal air pollution exposure.

**Citation:**

Breton CV, Yao J, Millstein J, Gao L, Siegmund KD, Mack W, Whitfield-Maxwell L, Lurmann F, Hodis H, Avol E, Gilliland FD. 2016. Prenatal air pollution exposures, DNA methyl transferase genotypes, and associations with newborn LINE1 and Alu methylation and childhood blood pressure and carotid intima-media thickness in the Children’s Health Study. Environ Health Perspect 124:1905–1912; http://dx.doi.org/10.1289/EHP181

## Introduction

Exposure to ambient air pollutants is a risk factor for cardiovascular disease (CVD) in adults (Jalaludin and Cowie 2014; Newby et al. 2015). Long-term exposures have been associated with measures of atherosclerosis, including carotid intima-media thickness (CIMT) and blood pressure (BP), both of which predict future cardiovascular events (Bianchini et al. 2013; Chirinos 2012; Vos et al. 2003). To our knowledge, air pollutant exposures early in life, particularly during the prenatal period, have not been evaluated for their contribution to CVD risk in humans, although this hypothesis is supported by the developmental origins of health and disease (DOHad) theory and increasing data from animal models (Gillman et al. 2007; Kelishadi and Poursafa 2014).

Epigenetic mechanisms may mediate prenatal exposures and CVD risk later in life (Kelishadi and Poursafa 2014; Whayne 2015; Zaina et al. 2014). Animal models provide evidence that prenatal exposures to environmental insults can alter epigenetic mechanisms in the offspring (Perera and Herbstman 2011; Skinner 2015) including altered epigenetic drift at transposon-associated metastable loci (Faulk et al. 2014). In humans, retrotransposons comprise roughly 50% of the human genome (Lander et al. 2001); however, the majority of these are thought to be transcriptionally inactive (Xing et al. 2007). During embryogenesis, transcriptional activities of the common elements LINE1 (long interspersed nuclear elements) and Alu increase markedly for a short period of time following genome-wide demethylation at the 2-cell stage but decrease back to the basal level before genome-wide remethylation and are thought to be maintained at a relatively constant hypermethylated state thereafter in somatic cells (Guo et al. 2014; Smith et al. 2014). This brief activity early in embryogenesis may lead to the creation of human-specific transcription factor binding sites associated with a pluripotent stem cell phenotype (Glinsky 2015). Therefore, altered DNA methylation during this critical embryonic stage may have broad potential to affect downstream regulatory function in the fetus. Given the relatively constant state of hypermethylation of LINE1 postimplantation (Smith et al. 2014), we would predict that any environmentally induced alterations to LINE1 during embryogenesis would be perpetuated throughout fetal development and could therefore be detected at birth in tissue such as newborn blood.

In a longitudinal study of > 700 elderly men, recent exposures to black carbon and PM_2.5_ (particulate matter with aerodynamic diameter ≤ 2.5 μm) were significantly associated with hypomethylation of LINE1 in blood leukocytes (Baccarelli et al. 2009). However, data on the effects of prenatal exposures on LINE1 methylation at birth or later in life are sparse. To our knowledge, only one study has reported the effects of prenatal air pollution on global DNA methylation. In a Belgian birth cohort (*n* = 240), first-trimester exposure to PM_2.5_ was significantly associated with lower global DNA methylation in placental tissues (Janssen et al. 2013). Understanding mechanisms through which air pollutants might alter methylation of repetitive DNA sequences such as Alu and LINE1, and determining whether there are time points of greatest susceptibility, is important because these elements have been associated with genomic instability and cancer (Belancio et al. 2010), higher systolic, diastolic, and mean arterial blood pressures, and increased risk of stroke (Baccarelli et al. 2010b; Kim et al. 2010).

Several genes that encode methyl transferase and demethylases play pivotal roles in the dynamic processes of methylation during embryogenesis. These include DNA methyl transferases (DNMTs: *DNMT1, DNMT3A* and *3B*) (Goll and Bestor 2005), the *TET* (ten-eleven translocation) family of enzymes (*TET 1, 2, 3*) (Kohli and Zhang 2013), and *TDG* (thymine DNA glycosylase) (Hu et al. 2014; Kohli and Zhang 2013). Thus, we hypothesized that variants in these genes may alter an individual’s inherent ability to methylate or demethylate LINE1 and AluYb8 elements during embryonic development. Because experimental data demonstrate that particulate matter can affect DNA methyltransferase activities (Wang et al. 2012), we hypothesized that air pollutant exposures and polymorphisms in DNA methyl transferase and demethylases may jointly affect individual susceptibility to later cardiovascular phenotypes in children. Therefore, we tested our hypotheses in the following steps: *a*) We investigated the association between prenatal air pollutants and cardiovascular phenotypes in childhood; *b*) we tested whether prenatal air pollutants were associated with different levels of LINE1 and AluYb8 methylation; *c*) we tested whether LINE1 or AluYb8 methylation at birth predicted cardiovascular phenotypes at age 11 years; and *d*) we investigated whether polymorphisms in DNA methyl transferase and demethylases modified these associations.

## Subjects and Methods

### Study Population

In this study we investigated the relationships between prenatal air pollutant exposures, LINE1 and AluYb8 methylation levels, and cardiovascular phenotypes in 459 participants who were recruited from nonsmoking households to participate in a substudy of atherosclerosis nested within the Children’s Health Study (Dratva et al. 2013; McConnell et al. 2006) and on whom DNA methylation was successfully measured. Children were first enrolled in kindergarten or first grade from public schools in 13 Southern California communities during the 2002–2003 school year and followed yearly. Communities were selected to represent the range and mixture of regional particulate pollutants, nitrogen dioxide (NO_2_), and ozone (O_3_) in Southern California. During a classroom visit when participants were 11 years old on average, systolic/diastolic BP, supine heart rate, and B-mode carotid artery ultrasound were performed. DNA methylation was measured in newborn blood spot tests that were obtained from the California Department of Public Health Genetic Disease Screening Program. Personal, parental, and sociodemographic characteristics were obtained by parent-completed questionnaire each year. *In utero* tobacco smoke exposure was assessed by asking whether the mother had ever smoked while pregnant with this child. Birth weight, gestational age, mode of delivery, and other reproductive data were obtained from California birth records. The estimated date of conception was assigned using the birth date and gestational age as indicated on the birth certificate, corrected for the average 2-week difference between the last menstrual period and conception.

The institutional review board for human studies at the University of Southern California approved the study protocol, and parents or legal guardians gave informed consent for all study participants.

### Air Pollution Assessment

The CHS air quality monitoring data (Peters et al. 1999a, 1999b) and the U.S. Environmental Protection Agency (EPA) Air Quality System (AQS; https://www.epa.gov/aqs) were used to assign estimates of prenatal air pollution exposures for PM_2.5_, PM_10_ (PM with aerodynamic diameter ≤10 μm), NO_2_, and O_3_, based on a combination of residential history obtained from parents when participants were 6–7 years old and birth address recorded on the birth certificate. In all but 34 cases, questionnaire-reported birth address from the residential history matched the birth address from the birth certificate. In the 34 cases where a mismatch was identified, the birth certificate address was used to assign air pollution exposure. Moreover, the listed birth address was reported to be the only residence during pregnancy for 88% of the participants.

Addresses were geocoded using TeleAtlas Inc.’s Address Point Geocoding Services. Station-specific air quality data were spatially interpolated to each birth residence using inverse-distance-squared weighting. The data from up to four air quality measurement stations were included in each interpolation. Due to the regional nature of O_3_, NO_2_, PM_10_, and PM_2.5_ concentrations, a maximum interpolation radius of 50 km was used for all pollutants. However, when a residence was located within 5 km of one or more stations with observations, the interpolation was based solely on the nearby values. Prenatal ambient air pollution concentrations were estimated by trimester for each subject’s birth residence with trimesters defined as follows: first trimester from 0 to 13 weeks postconception, second trimester from 14 to 26 weeks, and third trimester from 27 weeks to delivery.

### Health Measurements

High-resolution B-mode ultrasound images of the right and left common carotid arteries were obtained with a portable Biosound MyLab 25 ultrasound system attached to a 10-MHz linear array transducer by a single technician using standardized imaging and processing protocols as previously described (Hodis et al. 2001, 2002; Selzer et al. 1994, 2001). The jugular vein and carotid artery were imaged transversely with the jugular vein stacked above the carotid artery. All images contained internal anatomical landmarks for reproducing probe angulation and a single-lead electrocardiogram was recorded simultaneously with the B-mode image to ensure that CIMT was measured at the R-wave in the cardiac cycle. CIMT was measured along the far (deep) wall of the distal common carotid artery (0.25 cm from the carotid artery bulb) along a standard 1-cm length that was automatically determined by a computer-generated ruler with an in-house developed software package (Hodis et al. 2001, 2002; Selzer et al. 1994, 2001). This method standardizes the timing, location, and distance over which CIMT is measured, ensuring comparability across participants. Duplicate scans were conducted 2.5 days apart on average for CIMT (*n* = 44) and yielded intraclass correlation coefficients of 0.88 and 0.84 for left and right CIMT, respectively.

### DNA Methylation

DNA methylation was measured in archived newborn blood spots as the most proximal biomarker of DNA methylation reflecting the fetal experience. Laboratory personnel performing DNA methylation analysis were blinded to study subject information. Newborn blood spots were stored by the state of California at –20°C. Upon receipt, the blood spots were stored in the Southern California Environmental Health Sciences molecular biology laboratory at –80°C until DNA extraction. DNA was extracted using the Qiagen QiaAmp DNA micro kit (Qiagen, Inc.). Bisulfite modification of 300 ng genomic DNA was performed by using the EZ-96 DNA Methylation-Gold Kit (Zymo Research). Methylation analyses of LINE1 and AluYb8 were performed by bisulfite polymerase chain reaction (PCR) pyrosequencing assay using the HotMaster Mix (Eppendorf) and the PSQ HS 96 Pyrosequencing System (Biotage AB). LINE1 had four CpG loci, only three of which had similar average methylation values. Only the three correlated CpGs with high levels of methylation were averaged together and evaluated. AluYb8 also had three loci with correlated high mean levels of methylation. These were also averaged together as a summary metric. DNA was first used for LINE1 analysis. Remaining DNA was used for AluYb8 analysis, resulting in fewer participants with measured AluYb8.

### SNP Selection and Genotyping

Two hundred seventy-seven SNPs (single-nucleotide polymorphisms) within the range of 20 kb up- or downstream of the following genes were genotyped: *DNMT1, DNMT3A, DNMT3B, DNMT3L, TET1, TET2, TET3, TDG* using the Illumina HumanHap550, HumanHap550-Duo or Human610-Quad BeadChip microarrays as described previously (Torgerson et al. 2011). Fifteen SNPs were removed due to MAF (minor allele frequency) < 1%, resulting in 262 SNPs for analysis. Data were phased using SHAPEIT (https://mathgen.stats.ox.ac.uk/genetics_software/shapeit/shapeit.html#citations; Delaneau and Marchini 2014) and imputed using IMPUTE2 (https://mathgen.stats.ox.ac.uk/impute/impute_v2.html) with 1000Genomes Phase 1 integrated variant v3 phased reference (April 2012). RS numbers (reference SNP ID numbers), minor allele frequencies, and genomic location are shown in Excel File S1. Admixture was assessed using the program STRUCTURE from a set of ancestral informative markers that were scaled to represent the proportion of African-American, Asian, Native American, and white admixture (Pritchard et al. 2000). Admixture was assessed as a way to adjust for genetic ancestry that might capture additional information not captured by self-reported race/ethnicity.

### Statistical Analysis

Descriptive analyses examined the distribution of methylation by subject characteristics and trimester-specific air pollutant exposure groups. Spearman correlations of pollutants both between and within each trimester were calculated. Air pollutant exposures were scaled to a 2–standard deviation range within each trimester and the same trimester-specific contrast was used in all models.

To estimate associations between trimester-specific air pollutant exposures and LINE1 methylation or cardiovascular phenotypes (CIMT and BP) and to estimate associations between LINE1 and AluYb8 methylation and cardiovascular phenotypes, we fitted linear regression models for each pollutant individually adjusted for sex, admixture, mother’s education level (in five categories: less than 12th grade, completed grade 12, some college or technical school, completed 4 years of college, and some graduate training after college), *in utero* tobacco smoke (self-report, did mother ever smoke during pregnancy), and plate effect (for LINE1 analyses only). These covariates were chosen given *a priori* hypotheses that they may be associated with DNA methylation levels and were assessed when the participant was 11 years old. Additional adjustment for paternal education level and season of birth (defined as warm season if baby was born between April and September, or cool season otherwise) did not change the effect estimates by > 10% and were removed from final models. Complete case analyses were performed. Because it was not normally distributed, AluYb8 methylation level was dichotomized based on median value and its association with air pollutant exposure was tested using a logistic regression model. The smaller AluYb8 sample set had no participants with *in utero* smoke exposure, and all samples were measured in one plate, so the models did not include those covariates. We also evaluated the effect of multiple pollutants on LINE1 and AluYb8 DNA methylation by including all four pollutants within each trimester in one model.

We estimated the associations between variants in eight genes: *DNMT1, DNMT3A, DNMT3B, DNMT3L, TET1, TET2, TET3,* and *TDG,* and LINE1 and AluYb8 methylation using linear regression models described above. All genes were initially coded as ordinal variables to indicate the number of alleles (in which 0 coded the minor allele, 1 the heterozygote, and 2 the major allele). To estimate the joint effects of SNPs and pollutants on methylation, we included an interaction term between SNP and air pollution exposure in the regression models. Wald’s test was used to derive interaction *p*-values, and false discovery rate (FDR) was used to adjust for multiple testing. To generate figures, models were stratified by genotype. AluYb8 was not further analyzed given the small sample size. SNPs that demonstrated significant interactions (FDR-adjusted *p*-values < 0.05) in these models were then evaluated for interaction in the linear regression models evaluating the associations between air pollutants and cardiovascular phenotypes.

## Results

Prenatal air pollution assignments were successfully made for 442 of the 459 participants, except for PM_2.5_, for which we had only 318 participants with assigned exposure due to lack of monitoring data in some communities. Removal of participants with incomplete information for LINE1 methylation or modeled covariates yielded a complete analytic data set of 392 participants for O_3_, NO_2_, and PM_10._ There were 302 participants for PM_2.5_. For AluYb8 there were 181 participants available for analysis of O_3_, NO_2_, and PM_10_ and 140 available for PM_2.5_.

Of the 392 children in this study, 46% were male, 53% were of Hispanic white ethnicity, and 3% were exposed *in utero* to smoke (Table 1). LINE1 and AluYb8 each represent the average of 3 highly methylated and correlated CpG loci (see Figure S1). LINE1 methylation was normally distributed with a mean (± SD) of 74.4 ± 3.1%. AluYb8 methylation was negatively skewed with a median [interquartile range (IQR)] of 90.0% (1.3%). Distributions of ambient air pollutants are shown in Figure S2 by trimester and represent a wide range of exposure, as expected in Southern California. Pollutants were weakly to moderately correlated (see Table S2), with O_3_ and PM_10_ showing the strongest correlations within trimesters (correlations ranged from 0.65 to 0.71).

**Table 1:** Sociodemographic and cardiovascular characteristics of study participants at age 11 (*n* = 392).

Variable	*n*^*a*^	% or mean ± SD	Median	Minimum	Maximum	IQR
Male	181	46
Race/ethnicity
Non-Hispanic white	135	34
Hispanic white	208	53
Other race	49	13
*In utero* smoke	10	3
Maternal education level
Less than high school	53	14
Completed high school	65	17
Some college or technical school	144	37
Completed 4-year college	64	16
Graduate training post college	66	17
Age (years)	392	11.2 ± 0.6	11.2	9.9	12.8	0.9
Birth weight (g)	392	3413.6 ± 519.4	3430.0	709.0	5216.0	632.0
Right CIMT (mm)	392	0.565 ± 0.043	0.566	0.437	0.734	0.062
Left CIMT (mm)	392	0.561 ± 0.047	0.563	0.361	0.701	0.059
Diastolic blood pressure (mmHg)	392	57.0 ± 6.0	57.0	40.0	84.0	8.0
Systolic blood pressure (mmHg)	392	104.4 ± 8.3	105.0	85.0	128.0	11.0
LINE1 Methylation % in NBS	392	74.4 ± 3.1	74.3	64.3	93.5	2.4
AluYb8 Methylation % in NBS	181	89.5 ± 1.8	90.0	79.4	92.7	1.3
Abbreviations: IQR, interquartile range; NBS, newborn blood spot. ^***a***^AluYb8 is the only variable with missing data.						

When we evaluated the overall association between a 2-SD increase in each prenatal pollutant and cardiovascular phenotypes measured at 11 years of age, most trimester-specific air pollutant exposures were not significantly associated with outcomes (Table 2). However, a 21-ppb increase in third-trimester exposure to NO_2_ was significantly associated with a 2.33-mmHg [95% confidence interval (CI): 0.51, 4.15] higher BP in childhood.

**Table 2:** Association between a 2-SD change in air pollutants and carotid intima-media thickness and blood pressure, by trimester (*n* = 392).

Trimester	PM_2.5_^*a*^ (μg/m^3^)		PM_10_ (μg/m^3^)		NO_2_ (ppb)		O_3_ (ppb)	
	β^*b*^ (95% CI)	*p*-Value	β^*b*^ (95% CI)	*p*-Value	β^*b*^ (95% CI)	*p*-Value	β^*b*^ (95% CI)	*p*-Value
1st
Right CIMT (mm)	–0.002 (–0.01, 0.01)	0.71	0.001 (–0.01, 0.01)	0.90	–0.01 (–0.01, 0.003)	0.18	–0.001 (–0.01, 0.01)	0.79
Left CIMT (mm)	–0.001 (–0.01, 0.01)	0.81	–0.004 (–0.01, 0.01)	0.43	0.002 (–0.01, 0.01)	0.66	–0.003 (–0.01, 0.01)	0.57
Diastolic BP (mmHG)	0.79 (–0.63, 2.22)	0.28	–0.01 (–1.23, 1.21)	0.99	0.55 (–0.68, 1.79)	0.38	–0.66 (–1.91, 0.58)	0.30
Systolic BP (mmHG)	0.92 (–0.99, 2.82)	0.35	–0.61 (–2.27, 1.06)	0.47	0.77 (–0.92, 2.46)	0.37	–0.61 (–2.31, 1.09)	0.48
2nd
Right CIMT (mm)	–0.001 (–0.01, 0.01)	0.83	0.003 (–0.01, 0.01)	0.54	–0.003 (–0.01, 0.01)	0.55	0.004 (–0.01, 0.01)	0.36
Left CIMT (mm)	–0.003 (–0.01, 0.01)	0.64	–0.001 (–0.01, 0.01)	0.84	0.01 (–0.01, 0.02)	0.34	0.004 (–0.01, 0.01)	0.39
Diastolic BP (mmHG)	–0.82 (–2.25, 0.61)	0.26	–0.09 (–1.3, 1.13)	0.89	0.40 (–0.95, 1.74)	0.56	–0.17 (–1.39, 1.04)	0.78
Systolic BP (mmHG)	–0.13 (–2.04, 1.78)	0.89	–0.47 (–2.12, 1.19)	0.58	1.47 (–0.36, 3.29)	0.12	0.22 (–1.43, 1.87)	0.80
3rd
Right CIMT (mm)	–0.01 (–0.02, 0.004)	0.22	–0.002 (–0.01, 0.01)	0.71	–0.001 (–0.01, 0.01)	0.88	–0.01 (–0.01, 0.004)	0.30
Left CIMT (mm)	–0.01 (–0.02, 0.01)	0.40	–0.004 (–0.01, 0.01)	0.43	0.01 (–0.01, 0.02)	0.34	–0.004 (–0.01, 0.01)	0.43
Diastolic BP (mmHG)	0.02 (–1.42, 1.46)	0.98	0.32 (–0.89, 1.53)	0.61	0.94 (–0.40, 2.28)	0.17	0.26 (–0.99, 1.52)	0.68
Systolic BP (mmHG)	0.97 (–0.95, 2.90)	0.32	0.83 (–0.82, 2.48)	0.32	2.33 (0.51, 4.15)	0.01	0.18 (–1.53, 1.89)	0.84
^***a***^*n* = 302. ^***b***^Models are adjusted for sex, age at CIMT, *in utero* smoke, maternal education, and admixture. The 2 SD for the 1st-trimester pollutants PM_2.5_, PM_10_, NO_2_, and O_3_ are 14 μg/m^3^, 32 μg/m^3^, 21 ppb, and 44 ppb, respectively. The 2 SD for the 2nd-trimester pollutants PM_2.5_, PM_10_, NO_2_, and O_3_ are 15 μg/m^3^, 33 μg/m^3^, 21 ppb, and 43 ppb, respectively. The 2 SD for the 3rd-trimester pollutants PM_2.5_, PM_10_, NO_2_, and O_3_ are 12 μg/m^3^, 30 μg/m^3^, 21 ppb, and 39 ppb, respectively.								

When we next evaluated DNA methylation in repetitive elements, first-trimester PM_10_ and O_3_ pollutants were associated with lower LINE1 methylation in single pollutant models (Table 3). A 2-SD increase in PM_10_ (32 μg/m^3^) and O_3_ (43 ppb) exposures was associated with lower LINE1 methylation (β = –0.66; 95% CI: –1.22, –0.09 and β = –0.86; 95% CI: –1.42, –0.30, respectively) after adjustment for covariates (Table 3). Third-trimester O_3_ was associated with higher LINE1 methylation (β = 0.60; 95% CI: 0.01, 1.19). No associations were observed for AluYb8; however, these analyses were limited to only 181 children, and AluYb8 was dichotomized above or below the median (Table 3).

**Table 3:** The association between a 2 SD change in pollutants and LINE1 (*n* = 392) and AluYb8 (*n* = 181) methylation percentage, by trimester.

Trimester	LINE1		AluYb8 (high vs low)	
	β^*b*^ (95% CI)	*p*-Value	OR^*b*^ (95% CI)	*p*-Value
1st
PM_2.5_^*a*^	–0.55 (–1.22, 0.11)	0.10	1.02 (0.51, 2.01)	0.97
PM_10_	–0.66 (–1.22, –0.09)	0.02	1.00 (0.54, 1.86)	1.00
NO_2_	–0.33 (–0.88, 0.23)	0.25	1.76 (0.92, 3.35)	0.09
O_3_	–0.86 (–1.42, –0.30)	0.003	0.77 (0.41, 1.43)	0.40
2nd
PM_2.5_^*a*^	–0.33 (–1.00, 0.34)	0.33	0.71 (0.37, 1.38)	0.31
PM_10_	–0.17 (–0.74, 0.40)	0.55	0.83 (0.44, 1.60)	0.58
NO_2_	–0.28 (–0.86, 0.30)	0.34	1.09 (0.57, 2.08)	0.80
O_3_	0.22 (–0.35, 0.79)	0.45	0.82 (0.44, 1.52)	0.52
3rd
PM_2.5_^*a*^	0.42 (–0.29, 1.13)	0.24	0.87 (0.42, 1.80)	0.70
PM_10_	0.21 (–0.36, 0.78)	0.47	0.83 (0.45, 1.52)	0.54
NO_2_	0.001 (–0.60, 0.61)	1.00	0.95 (0.47, 1.90)	0.88
O_3_	0.60 (0.01, 1.19)	0.05	1.06 (0.57, 1.97)	0.86
^***a***^*n* = 302 for LINE1 and 140 for AluYb8. ^***b***^Models were adjusted for admixture, sex, plate, *in utero* tobacco smoke (LINE1 only) and maternal education level. The 2 SD for the 1st-trimester pollutants PM_2.5_, PM_10_, NO_2_, and O_3_ are 14 μg/m^3^, 32 μg/m^3^, 21 ppb, and 44 ppb, respectively. The 2 SD for the 2nd-trimester pollutants PM_2.5_, PM_10_, NO_2_, and O_3_ are 15 μg/m^3^, 33 μg/m^3^, 21 ppb, and 43 ppb, respectively. The 2 SD for the 3rd-trimester pollutants PM_2.5_, PM_10_, NO_2_, and O_3_ are 12 μg/m^3^, 30 μg/m^3^, 21 ppb, and 39 ppb, respectively.				

In multi-pollutant models in which all four pollutants were mutually adjusted, we found that the associations with NO_2_ and PM_10_ were most robust (see Table S2). After controlling for other pollutant levels, a 43-ppb increase in second- and a 39-ppb increase in third-trimester O_3_ were significantly associated with higher LINE1 methylation levels (β = 0.94; 95% CI: 0.08, 1.80 and β = 1.06; 95% CI: 0.16, 1.95, respectively). No significant associations were observed for AluYb8 analyses, although the sample size was smaller (*n* = 140) (see Table S2). A 1% increase in LINE1 and AluYb8 methylation levels was not significantly associated with cardiovascular phenotypes (see Table S3).

**Figure 1:**
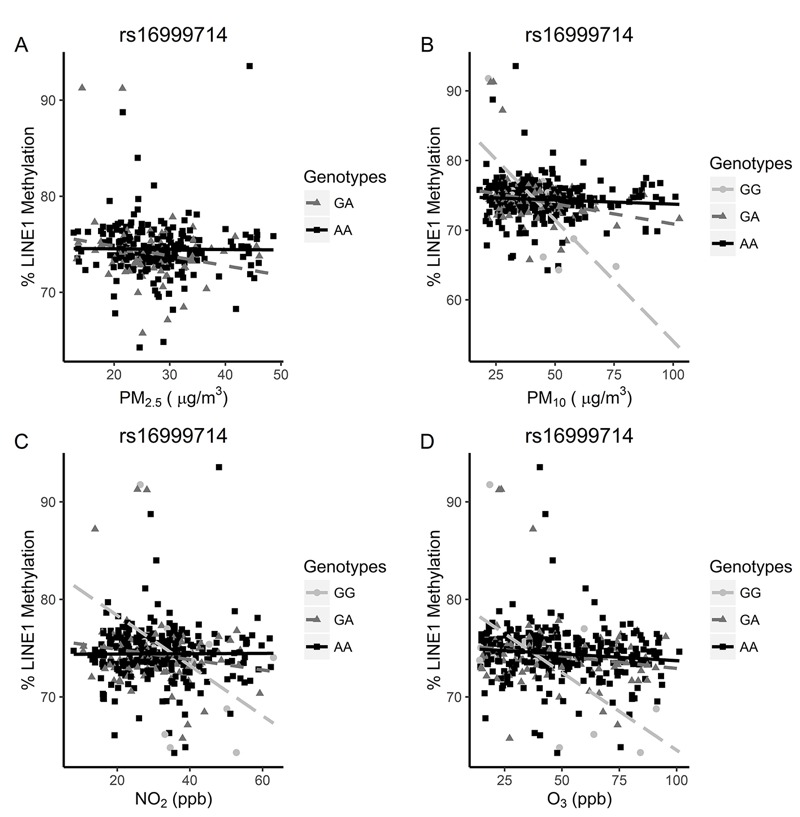
The association between 1st-trimester air pollutants (*A*) PM_2.5_, (*B*) PM_10_, (*C*) NO_2_, and (*D*) O_3_ and LINE1 methylation percentage by DNMT1 rs16999714 genotype. Complete data are available in Table 5. The 2 SDs for 1st-trimester pollutants PM_2.5_, PM_10_, NO_2_, and O_3_ are 14 μg/m^3^, 32 μg/m^3^, 21 ppb, and 44 ppb, respectively.

Because genetic variation in methyl transferase and demethylases may directly alter the dynamic processes of methylation, we further investigated whether 262 SNPs in or near *DNMT1, DNMT3A, DNMT3B, DNMT3L, TET1, TET2, TET3,* and *TDG* altered the associations between first-trimester air pollutants and LINE1 methylation. Although none of the SNPs were directly associated with LINE1 methylation level (data not shown), 11 unique SNPs in four genes demonstrated significant interactions with at least one first-trimester air pollutant and LINE1 methylation after FDR adjustment for multiple testing (Table 4). The full list of results for interaction test *p*-values between air pollutants and SNPs on LINE1 methylation is shown in Excel File S2. One of these SNPs had significant interactions with all four air pollutants and is highlighted in Figure 1. Associations between LINE1 DNA methylation and first-trimester PM_10_, NO_2_, and O_3_ exposures were stronger in children with the homozygous recessive GG genotype for rs16999714 in *DNMT1* than in individuals with other genotypes (Figure 1). For example, a 32-μg/m^3^ higher first-trimester PM_10_ was associated with a 23.6% (95% CI: –38.4%, –8.8%) lower LINE1 DNA methylation level in individuals with GG genotype, compared with no effect in individuals with the GA (β = 1.2%; 95% CI: –2.7%, 0.2%) or AA (β = –0.2; 95% CI: –0.8, 0.3; *p*-value_int_ = 0.0002) genotypes (Figure 1; Table 5). However, these associations were based on only 14 individuals with the GG genotype and should therefore be interpreted with caution.

**Table 4:** SNPs and 1st-trimester air pollutants that were jointly associated with LINE1 methylation %.*^a^*

Gene ± 20 kb	RS Number	Chr	Location	Pollutant	β_pollutant_ (95% CI)	β_SNP_ (95% CI)	β_int_ (95% CI)	FDR *p*-value_int_	*n*	Chromatin state in embryonic stem cells^*b*^
*DNMT1*	rs16999714	19	10177450	NO_2_	–3.02 (–4.79, –1.25)	–2.24 (–3.93, –0.55)	1.59 (0.60, 2.58)	0.04	388	Enhancer/quiescent
*DNMT1*	rs16999714	19	10177450	O_3_	–4.00 (–5.71, –2.28)	–1.58 (–2.74, –0.41)	1.85 (0.87, 2.83)	0.01	388	Enhancer/quiescent
*DNMT1*	rs16999714	19	10177450	PM_10_	–5.22 (–7.25, –3.18)	–3.10 (–4.67, –1.52)	2.60 (1.48, 3.72)	0.0002	388	Enhancer/quiescent
*DNMT1*	rs16999714	19	10177450	PM_2.5_	–4.13 (–6.29, –1.97)	–3.49 (–5.93, –1.04)	2.10 (0.88, 3.32)	0.02	298	Enhancer/quiescent
*DNMT3B*	rs17123673	20	30873266	PM_2.5_	8.35 (3.97, 12.74)	8.72 (4.16, 13.27)	–4.66 (–6.93, –2.39)	0.002	302	Enhancer/weak transcription
*DNMT3B*	rs20654	20	30881957	PM_2.5_	1.50 (0.15, 2.86)	3.39 (1.44, 5.33)	–1.68 (–2.64, –0.71)	0.02	298	Weak/strong transcription
*DNMT3B*	rs6579038	20	30894431	O_3_	–7.48 (–11.44, –3.52)	–4.57 (–7.07, –2.08)	3.42 (1.39, 5.45)	0.03	390	Strong transcription
*DNMT3B*	rs6579038	20	30894431	PM_10_	–7.80 (–11.87, –3.73)	–5.45 (–8.33, –2.57)	3.70 (1.62, 5.78)	0.01	390	Strong transcription
*DNMT3B*	rs8121782	20	30893499	PM_10_	–7.66 (–11.80, –3.52)	–5.42 (–8.35, –2.49)	3.61 (1.49, 5.73)	0.02	392	Weak/strong transcription
*TDG*	rs3794240	12	102853671	PM_10_	–3.90 (–5.73, –2.07)	–2.79 (–4.29, –1.28)	1.97 (0.90, 3.04)	0.01	385	Strong transcription
*TDG*	rs4135036	12	102883184	PM_2.5_	–7.17 (–11.11, –3.23)	–8.37 (–12.54, –4.2)	3.48 (1.43, 5.53)	0.04	302	Active TSS
*TET2*	rs2726459	4	106404046	PM_2.5_	1.35 (0.16, 2.54)	3.74 (1.78, 5.70)	–1.89 (–2.89, –0.90)	0.01	301	No data
*TET2*	rs4698932	4	106268596	PM_2.5_	1.89 (0.60, 3.18)	4.47 (2.43, 6.52)	–2.24 (–3.27, –1.21)	0.001	265	Quiescent
*TET2*	rs7655049	4	106271913	PM_2.5_	4.78 (2.39, 7.16)	5.62 (3.01, 8.23)	–3.04 (–4.35, –1.73)	0.0003	299	Quiescent
*TET2*	rs7678440	4	106398351	PM_2.5_	1.42 (0.25, 2.58)	3.97 (2.01, 5.93)	–2.03 (–3.03, –1.03)	0.004	302	No data
Chr, chromosome; RS, reference SNP; TSS, transcription start sites. ^***a***^SNPs were modeled as ordinal variables (in which 0 = minor allele, 1 = heterozygote, and 2 = major allele), and models were adjusted for admixture, sex, plate, *in utero* tobacco smoke, and maternal education level. The 2 SD for the 1st-trimester pollutants PM_2.5_, PM_10_, NO_2_, and O_3_ are 14 μg/m^3^, 32 μg/m^3^, 21 ppb, and 44 ppb, respectively. ^***b***^Derived from RegulomeDB (Boyle et al. 2012).										

**Table 5:** Association between a 2-SD increase in 1st-trimester pollutants and LINE1 methylation percentage by DNMT1 rs16999714 genotype.

Pollutant	Genotype	*n*	β^*a*^ (95% CI)	*p*-Value	*p*-Value_int_
PM_2.5_	GG	11	NA^*b*^	NA^*b*^	0.02
PM_2.5_	GA	75	–2.0 (–3.7, –0.4)	0.02
PM_2.5_	AA	212	0.0 (–0.6, 0.7)	0.90
PM_10_	GG	14	–23.6 (–38.4, –8.8)	0.04	0.0002
PM_10_	GA	92	–1.2 (–2.7, 0.2)	0.10
PM_10_	AA	282	–0.2 (–0.8, 0.3)	0.41
NO_2_	GG	14	–12.4 (–25.8, 1.0)	0.14	0.04
NO_2_	GA	92	–1.3 (–2.7, 0.2)	0.08
NO_2_	AA	282	0.2 (–0.4, 0.8)	0.52
O_3_	GG	14	–15.8 (–24.0, –7.7)	0.02	0.005
O_3_	GA	92	–0.8 (–2.1, 0.5)	0.21
O_3_	AA	282	–0.5 (–1.1, 0.1)	0.09
^***a***^Models were adjusted for admixture, sex, plate, *in utero* tobacco smoke, and maternal education level. The 2 SD for the 1st-trimester pollutants PM_2.5_, PM_10_, NO_2_, and O_3_ are 14 μg/m^3^, 32 μg/m^3^, 21 ppb, and 44 ppb, respectively. ^***b***^Number of observations is less than that of parameters.					

**Figure 2:**
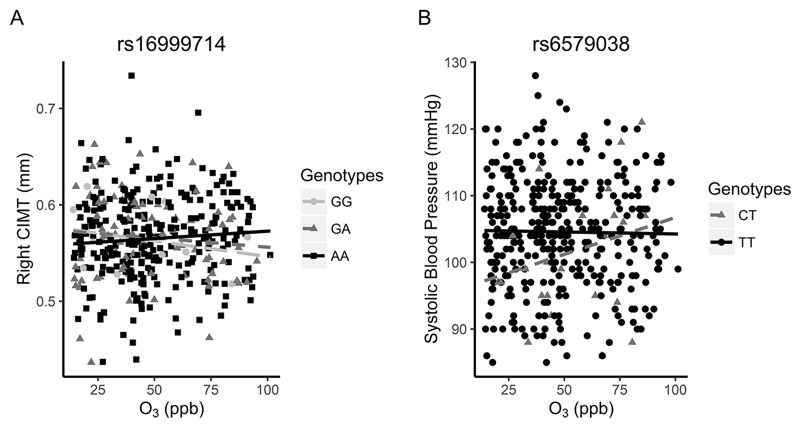
The association between first-trimester O_3_ and right CIMT by (*A*) rs16999714 and (*B*) rs6579038 genotypes. In *A*, a 44-ppb increase in O_3_ was associated with a –0.06-mm lower right CIMT (95% CI: –0.08, –0.04) in participants with the rs16999714 GG genotype (*n* = 14), a –0.02-mm lower right CIMT (95% CI: –0.03, 0.002) for participants with the GA genotype (*n* = 92), and a 0.005-mm higher right CIMT (95% CI: –0.006, 0.02) for participants with the AA genotype (*n* = 282). In *B*, a 44-ppb increase in O_3_ was associated with a 6.43-mmHg higher SBP (95% CI: –6.36, 19.21) in participants with the rs6579038 CT genotype (*n* = 25) and a 1.12-mmHg lower SBP (95% CI: –2.86, 0.61) in participants with the TT genotype (*n* = 364). There was no regression line for CC genotype, given that only one participant had this genotype.

Two of the 11 SNPs in Table 4 that modified associations between air pollutants and LINE1 methylation also modified associations between air pollutants and cardiovascular phenotypes measured in children at 11 years age, including right CIMT and BP (see Table S4). Specifically, a 44-ppb increase in O_3_ was associated with 0.005-mm higher (95% CI: –0.006, 0.02) right CIMT for individuals with the AA genotype for rs16999714, but a 0.02-mm lower (95% CI: –0.03, 0.002) right CIMT in individuals with the GA genotype and a 0.06-mm lower (95% CI: –0.08, –0.04) right CIMT in individuals with the GG genotype (Figure 2). A similar but not statistically significant pattern was seen with left CIMT (data not shown). A 44-ppb increase in O_3_ was associated with a 6.4-mmHg higher systolic BP among individuals with the CT genotype of rs6579038 but a 1.1-mmHg lower systolic BP in individuals with the TT genotype (Figure 2). There were too few individuals with the CC genotype to analyze (*n* = 1). These results suggest that the associations between air pollution exposure and cardiovascular phenotypes may depend partly on the population distribution of specific susceptibility loci in relevant genetic pathways active during the first trimester.

## Discussion

In this study, we found that NO_2_ exposure in the third trimester of pregnancy was associated with systolic BP in 11-year-old children. Although first-trimester O_3_ exposure was not associated with CIMT and systolic BP overall, associations within strata of DNMT1 were observed, and the magnitude and the direction of these associations depended on DNMT1 genotypes. Prenatal exposure to air pollutants in the first trimester was also associated with lower DNA methylation in LINE1, whereas third-trimester exposure to O_3_ was associated with higher LINE1 methylation levels in newborn blood spots. The magnitude of the associations between first-trimester air pollutants and LINE1 methylation depended on genetic sequence variation in *DNMT1, DNMT3B, TET2*, and *TDG* genes in the newborn.

Given our interest in the DOHad hypothesis related to CVD, we investigated whether prenatal exposure to air pollution was related to cardiovascular phenotypes in 11-year-old children. In adult populations, air pollutants are known risk factors for cardiovascular disease (Beelen et al. 2014; Jalaludin and Cowie 2014). Our observation that third-trimester NO_2_ exposure is associated with higher BP in children is in contrast with recent work showing that higher mean PM_2.5_ and black carbon but not NO_2_ exposures during the third trimester were associated with higher SBP at birth (van Rossem et al. 2015), although the timing of assessment differs by 11 years. Interestingly, in our study prenatal O_3_ exposure was associated with both CIMT and BP only when both *DNMT1* and *DNMT3B* genotypes were taken into consideration. This is in contrast with an overall positive association between O_3_ and CIMT that we observed previously in another population of young adults (Breton et al. 2012). Our present results suggest that discrepancies across studies in overall associations between pollutants and cardiovascular disease markers may be driven in part by lack of consideration for differences in genetic susceptibility.

Emerging evidence suggests that air pollution also affects DNA methylation levels in repetitive elements (Breton and Marutani 2014), and lower LINE1 methylation has been associated with various cardiovascular risk factors in the Normative Aging Study of elderly men (Baccarelli et al. 2010a, 2010b). In elderly men (*n* = 718), recent black carbon and PM_2.5_ exposures were associated with significantly lower LINE1 methylation (Baccarelli et al. 2009); and in a double-blind crossover study of 16 asthmatic adults, controlled exposure to diesel exhaust was associated with both increased and decreased methylation depending on the specific Alu and LINE1 CpG site evaluated (Baccarelli et al. 2009; Jiang et al. 2014). This research is in its infancy and little is known about the time periods of greatest susceptibility. Only a few studies have evaluated prenatal exposure to environmental pollutants with respect to repetitive element methylation. In one study of 380 women, placental AluYb8 levels were significantly higher in women who smoked during pregnancy, whereas LINE1 levels were significantly higher in women who drank alcohol during pregnancy (Wilhelm-Benartzi et al. 2012). Prenatal arsenic exposure was associated with small but significant increases in LINE1 methylation level, whereas prenatal lead was inversely associated with both LINE1 and Alu methylation (Kile et al. 2012; Pilsner et al. 2009). First-trimester exposure to PM_2.5_ was significantly associated with lower global DNA methylation in placental tissues from 240 women; however, no associations were observed for the second or third trimester (Janssen et al. 2013). This parallels our own findings, which suggest that the first trimester may be the most important window of exposure with respect to associations between PM_2.5_ and methylation, although we measured a specific repetitive element LINE1 and in newborn blood rather than placental tissue.

The conventional explanation for these observations is that oxidative DNA damage interferes with the capability of methyltransferases to interact with DNA, resulting in lower methylation of cytosine residues at CpG sites (Valinluck et al. 2004). After fertilization and before implantation, DNA methylation patterns are largely erased but are reestablished by *de novo* DNMTs in the blastocyst stage (Jirtle and Skinner 2007). Thus, this brief period of heightened transcriptional activity during DNA methylation reprogramming may be a period during which the fetus is particularly susceptible to harmful effects of environmental exposures such as air pollutants (Barouki et al. 2012).

During embryogenesis, transcriptional activities of LINE and Alu increase markedly for a short period of time following genome-wide demethylation at the two-cell stage but decrease back to the basal level before the genome-wide re-methylation (Guo et al. 2014). This pattern of activity contrasts with the conventional belief that transposable elements are highly methylated to prevent the potentially harmful effects of mutations on functional genome integrity and maintenance of genomic stability. New evidence suggests that transposable elements may contribute to the creation of human-specific transcription factor binding sites associated with a pluripotent stem cell phenotype (Glinsky 2015). If true, altered methylation during this critical stage may have broad potential to affect downstream regulatory function and presents a new hypothesis that has yet to be explored.

Because *DNMT*s, *TET*s, and *TDG* are all genes with distinct yet crucial roles to play in methylation reprogramming during embryogenesis (Dawlaty et al. 2014; Hu et al. 2014; Kohli and Zhang 2013), we investigated whether polymorphisms in these genes might alter the association between prenatal air pollutant exposures and LINE1 and AluYb8 methylation levels. We found this to be true most strongly for SNPs in *DNMT1*, but also for SNPs within or near *DNMT3B, TDG*, and *TET2*. None of the identified SNPs were exonic or in linkage disequilibrium with exonic SNPs. However, several SNPs were located in DNAse hypersensitive sites, putative enhancer locations, or promoters, and all of them had predicted changes in at least one binding motif according to HaploReg v2 (Ward and Kellis 2012).

The mechanisms by which variation in these genes might modify associations between air pollution and LINE1 methlyation are not currently understood. DNMT1 is primarily a maintenance methyltransferase that methylates hemimethylated DNA during DNA replication and *in vitro* (Kinney and Pradhan 2011). Studies of embryonic stem cells have demonstrated that deletion of *DNMT1* results in a substantial reduction in DNA methylation in many elements including LINE1 (Arand et al. 2012). Alternative isoforms of *DNMT1* have been identified which are expressed in pre-implantation embryos and early fetal development (Giraldo et al. 2013; Golding and Westhusin 2003). Thus, one explanation for our observed association is that polymorphisms in *DNMT1* affect expression of specific *DNMT1* isoforms active during embryogenesis at a time when LINE1 is also active, further decreasing function of the enzyme that may already be reduced under high air pollution scenarios, if pollution interferes with the *DNMT1* ability to bind DNA. Alternatively, these effects may be limited to certain cell types. For example, rs16999714 is located upstream of the DNMT1 transcription start site in a putative enhancer that appears active in H1 BMP4–derived trophoblast cells, adjacent to a poised enhancer in H1-derived mesenchymal stem cells but not in derived mesendoderm cells (see Figure S3) as well as other fetal tissues or cell lines (Ward and Kellis 2012; Zhou et al. 2011). This *in silico* evaluation suggests that the SNP may alter function of the gene in a specific subset of cells during embryogenesis.

Our results in *DNMT3B, TDG*, and *TET2* require further investigation as little is known about how they interact with pollutants. However, both *TDG* and *TET2* are essential for embryonic development, including induction of the mesoderm and hematopoietic differentiation (Langlois et al. 2014; Nakajima and Kunimoto 2014), maintenance of active and bivalent chromatin throughout cell differentiation, and initiation of base excision repair to counter aberrant *de novo* methylation (Cortázar et al. 2011).

This study has several limitations. Although we made every effort to control for potential confounders, we cannot exclude the possibility of residual confounding by some unknown factor that is associated with LINE1 or AluYb8 methylation levels, ambient air pollution, and cardiovascular phenotypes. Power for investigating AluYb8 was limited, particularly for interaction testing. Our study was not designed to evaluate temporal changes of DNA methylation. Therefore, DNA methylation measured in newborn blood spots may not reflect *in vivo* methylation patterns occurring earlier at critical points of development. Estimation of trimester-specific exposures based on birth certificate reporting of gestational age is prone to error (Dietz et al. 2007). Despite this inherent measurement error, we observed multiple associations primarily for first-trimester exposures. The present findings add to the emerging body of evidence that prenatal environmental exposures can be associated with LINE1 methylation; however, they should be replicated in additional populations. Future studies specifically designed to capture weekly exposures in pregnancy will also help to identify the relevant biological window of susceptibility.

## Conclusions

Prenatal exposure to multiple air pollutants was associated with DNA methylation in newborn LINE1 and the magnitude of the association depended on genetic variation in *DNMT1, DNMT3B, TET2*, and *TDG* genes. Genetic variation in two SNPs in DNMT genes also altered children’s susceptibility to prenatal O_3_-induced changes in cardiovascular phenotypes at 11 years of age. Both genetic and epigenetic variation in DNA methylation reprogramming genes and in LINE1 retrotransposons may play important roles in downstream cardiovascular consequences of prenatal air pollution exposure.

## Supplemental Material

(531 KB) PDF

(79 KB) ZIP
