# Supplementary material for: Prenatal Air Pollution Exposures, DNA Methyl Transferase Genotypes, and Associations with Newborn LINE1 and Alu Methylation and Childhood Blood Pressure and Carotid Intima-Media Thickness in the Children’s Health Study
Source: Environ Health Perspect. 2016 May 24;124(12):1905–12. doi: 10.1289/EHP181 (PMC5132634; doi:10.1289/EHP181)
Supplement: (531 KB) PDF [file EHP181.s001.acco.pdf]

Environ Health Perspect
DOI: 10.1289/EHP181

Note to readers with disabilities: *EHP* strives to ensure that all journal content is accessible to all readers. However, some figures and Supplemental Material published in *EHP* articles may not conform to 508 standards due to the complexity of the information being presented. If you need assistance accessing journal content, please contact ehp508@niehs.nih.gov. Our staff will work with you to assess and meet your accessibility needs within 3 working days.

Supplemental Material

Prenatal Air Pollution Exposures, DNA Methyl Transferase Genotypes, and Associations with Newborn LINE1 and Alu Methylation and Childhood Blood Pressure and Carotid Intima-Media Thickness in the Children's Health Study

Carrie V. Breton, Jin Yao, Josh Millstein, Lu Gao, Kimberly D. Siegmund, Wendy Mack, Lora Whitfield-Maxwell, Fred Lurmann, Howard Hodis, Ed Avol, and Frank D. Gilliland

Table of Contents

Table S1. Spearman correlation between trimester-specific pollutants (N=392)

Table S2. The association between LINE1 (N=302) and AluYb8 (N=140) methylation % and a 2 SD change in air pollutants using a single multi-pollutant model, by trimester

Table S3. Association between a 1 % increase in LINE1 (N=411) and AluYb8 (N=190) methylation and CIMT and blood pressure

Table S4. SNPs and 1st trimester air pollutants showing joint effects on cardiovascular phenotypes

Figure S1. Boxplots of percent methylation for each assayed CpG in a) LINE1 (N=392) and b) AluYb8 (N=181). Boxes extend from the 25th to the 75th percentile, horizontal bars represent the median, whiskers extend 1.5 times the length of the interquartile range (IQR) above and below the 75th and 25th percentiles, respectively, and outliers beyond whiskers are represented as points.

Figure S2. Distributions of ambient air pollutants by trimester (N=392). Boxes extend from the 25th to the 75th percentile, horizontal bars represent the median, whiskers extend 1.5 times the length of the interquartile range (IQR) above and below the 75th and 25th percentiles, respectively, and outliers are represented as points.

Figure S3. Illustration of rs16999714 (yellow bar) upstream of the DNMT1 transcription start site in a putative enhancer that appears a) inactive in H1 BMP4 derived mesendoderm cells, b) active in H1 BMP4 derived trophoblast cells, and c) adjacent to a poised enhancer in H1 derived mesenchymal stem cells. Blue is H3K27ac and green is H3K4me1. The figure was produced using the Wash U Epigenome Browser.

Reference

Additional Files

Supplemental Code and Data Zip File

Supplemental Code and Data Zip File Index

Excel File S1. List of 262 eligible SNPs analyzed

Excel File S2. Results for all SNP-1st trimester air pollutant interaction tests using a generalized linear regression model in which SNPs were treated as ordinal variables

Table S1. Spearman correlation between trimester-specific pollutants (N=392)

		T1				T2				T3			
		**$PM_{2.5}$[a]**	**PM_{10}**	**NO_2**	**O_3**	**$PM_{2.5}$[a]**	**PM_{10}**	**NO_2**	**O_3**	**$PM_{2.5}$[a]**	**PM_{10}**	**NO_2**	**O_3**
**T1**	**$PM_{2.5}$**	1.00	0.66*	0.48*	0.41*	0.19*	-0.21*	0.27*	-0.60*	-0.34*	-0.39*	-0.15	-0.24*
	**PM_{10}**		1.00	0.11	0.70*	0.32*	0.30*	0.04	-0.21*	-0.41*	-0.21*	-0.35*	-0.39*
	**NO_2**			1.00	0.01	-0.07	-0.42*	0.67*	-0.56*	-0.15	-0.38*	0.41*	-0.12
	**O_3**				1.00	0.61*	0.47*	0.22*	0.07	-0.44*	-0.50*	-0.20*	-0.65*
**T2**	**$PM_{2.5}$**					1.00	0.63*	0.39*	0.28*	-0.11	-0.37*	0.05	-0.56*
	**PM_{10}**						1.00	-0.01	0.65*	-0.01	0.11	-0.10	-0.29*
	**NO_2**							1.00	-0.25*	-0.14	-0.46*	0.60*	-0.41*
	**O_3**								1.00	0.31*	0.39*	0.01	0.09
**T3**	**$PM_{2.5}$**									1.00	0.58*	0.21*	0.46*
	**PM_{10}**										1.00	-0.03	0.71*
	**NO_2**											1.00	-0.12
	**O_3**												1.00

[a]N=302

*p<0.05

Table S2. The association between LINE1 (N=302) and AluYb8 (N=140) methylation % and a 2 SD change in air pollutants using a single multi-pollutant model, by trimester

	LINE1		AluYb8 (high vs low)	
**Pollutant**	**Estimate[a] (95% CI)**	**P-value**	**OR[a] (95%CI)**	**P-value**
**1st trimester**				
$PM_{2.5}$	0.60 (-0.53, 1.73)	0.30	0.43 (0.13, 1.42)	0.16
PM_{10}	-0.92 (-2.00, 0.15)	0.09	2.03 (0.66, 6.30)	0.21
NO_2	-0.85 (-1.81, 0.12)	0.08	2.48 (0.90, 6.89)	0.08
O_3	-0.52 (-1.35, 0.31)	0.22	0.85 (0.37, 1.95)	0.69
**2nd trimester**				
$PM_{2.5}$	0.18 (-0.92, 1.29)	0.74	0.33 (0.09, 1.12)	0.07
PM_{10}	-0.86 (-1.99, 0.26)	0.13	1.81 (0.51, 6.36)	0.35
NO_2	-0.32 (-1.42, 0.77)	0.56	2.49 (0.73, 8.50)	0.14
O_3	0.94 (0.08, 1.80)	0.03	1.00 (0.41, 2.46)	1.00
**3rd trimester**				
$PM_{2.5}$	0.13 (-0.86, 1.13)	0.79	0.84 (0.30, 2.39)	0.75
PM_{10}	-0.50 (-1.52, 0.53)	0.34	1.04 (0.38, 2.83)	0.94
NO_2	0.51 (-0.59, 1.60)	0.36	1.04 (0.32, 3.35)	0.95
O_3	1.06 (0.16, 1.95)	0.02	0.98 (0.41, 2.39)	0.97

[a] Models were adjusted for admixture, sex, plate, *in utero* tobacco smoke (LINE1 only) and maternal education level. The 2SD for the following 1st trimester pollutants $PM_{2.5}$, PM_{10}, NO_2, and O_3 are 14 µg/m^3, 32 µg/m^3, 21 ppb, and 44 ppb, respectively. The 2SD for the following 2nd trimester pollutants $PM_{2.5}$, PM_{10}, NO_2, and O_3 are 15 µg/m^3, 33 µg/m^3, 21 ppb, and 43 ppb, respectively. The 2SD for the following 3rd trimester pollutants $PM_{2.5}$, PM_{10}, NO_2, and O_3 are 12 µg/m^3, 30 µg/m^3, 21 ppb, and 39 ppb, respectively.

Table S3. Association between a 1 % increase in LINE1 (N=411) or AluYb8 (N=190) methylation and CIMT and blood pressure

	LINE1		AluYb8	
**Outcome**	**β[a] (95% CI)**	**p-value**	**β[a] (95% CI)**	**p-value**
Right CIMT (mm)	0.0004 (-0.0011, 0.0018)	0.63	0.0003 (-0.0031, 0.0038)	0.85
Left CIMT (mm)	0.0007 (-0.0009, 0.0023)	0.38	-0.0009 (-0.0046, 0.0028)	0.65
Diastolic blood pressure (mmHg)	-0.0407 (-0.2502, 0.1688)	0.70	-0.0676 (-0.5622, 0.4270)	0.79
Systolic blood pressure (mmHg)	-0.1259 (-0.4071, 0.1553)	0.38	-0.2164 (-0.8265, 0.3937)	0.49

[a]adjusted for sex, batch, age at CIMT, maternal smoking (LINE 1 only), maternal education and race/ethnicity

Table S4. SNPs and 1st trimester air pollutants showing joint effects on cardiovascular phenotypes[a]

Gene ± 20kb	RS Number	Chr	Location	Pollutant	CVD phenotype	P-value$_{int}$	N
DNMT1	rs16999714	19	10177450	NO_2	Systolic Blood Pressure (mmHg)	0.17	388
					Diastolic Blood Pressure (mmHg)	0.69	388
					Right CIMT (mm)	0.83	388
					Left CIMT (mm)	0.31	388
DNMT1	rs16999714	19	10177450	O_3	Systolic Blood Pressure (mmHg)	0.24	388
					Diastolic Blood Pressure (mmHg)	0.60	388
					Right CIMT (mm)	0.04	388
					Left CIMT (mm)	0.24	388
DNMT1	rs16999714	19	10177450	PM_{10}	Systolic Blood Pressure (mmHg)	0.61	388
					Diastolic Blood Pressure (mmHg)	0.21	388
					Right CIMT (mm)	0.68	388
					Left CIMT (mm)	0.41	388
DNMT1	rs16999714	19	10177450	$PM_{2.5}$	Systolic Blood Pressure (mmHg)	0.44	298
					Diastolic Blood Pressure (mmHg)	0.45	298
					Right CIMT (mm)	0.45	298
					Left CIMT (mm)	0.14	298
DNMT3B	rs17123673	20	30873266	$PM_{2.5}$	Systolic Blood Pressure (mmHg)	0.51	302
					Diastolic Blood Pressure (mmHg)	0.92	302
					Right CIMT (mm)	0.78	302
					Left CIMT (mm)	0.68	302
DNMT3B	rs20654	20	30881957	$PM_{2.5}$	Systolic Blood Pressure (mmHg)	0.76	298
					Diastolic Blood Pressure (mmHg)	0.77	298
					Right CIMT (mm)	0.42	298
					Left CIMT (mm)	0.85	298
DNMT3B	rs6579038	20	30894431	O_3	Systolic Blood	0.04	390

					Pressure (mmHg)		
					Diastolic Blood Pressure (mmHg)	0.09	390
					Right CIMT (mm)	0.81	390
					Left CIMT (mm)	0.29	390
DNMT3B	rs6579038	20	30894431	PM_{10}	Systolic Blood Pressure (mmHg)	0.10	390
					Diastolic Blood Pressure (mmHg)	0.20	390
					Right CIMT (mm)	0.41	390
					Left CIMT (mm)	0.18	390
DNMT3B	rs8121782	20	30893499	PM_{10}	Systolic Blood Pressure (mmHg)	0.09	392
					Diastolic Blood Pressure (mmHg)	0.19	392
					Right CIMT (mm)	0.38	392
					Left CIMT (mm)	0.14	392
TDG	rs3794240	12	102853671	PM_{10}	Systolic Blood Pressure (mmHg)	0.94	385
					Diastolic Blood Pressure (mmHg)	0.65	385
					Right CIMT (mm)	0.51	385
					Left CIMT (mm)	0.76	385
TDG	rs4135036	12	102883184	$PM_{2.5}$	Systolic Blood Pressure (mmHg)	0.08	302
					Diastolic Blood Pressure (mmHg)	0.24	302
					Right CIMT (mm)	0.75	302
					Left CIMT (mm)	0.79	302
TET2	rs2726459	4	106404046	$PM_{2.5}$	Systolic Blood Pressure (mmHg)	0.51	301
					Diastolic Blood Pressure (mmHg)	0.68	301
					Right CIMT (mm)	0.99	301
					Left CIMT (mm)	0.69	301
TET2	rs4698932	4	106268596	$PM_{2.5}$	Systolic Blood Pressure (mmHg)	0.33	265
					Diastolic Blood Pressure (mmHg)	0.83	265
					Right CIMT (mm)	0.73	265
					Left CIMT (mm)	0.57	265
TET2	rs7655049	4	106271913	$PM_{2.5}$	Systolic Blood Pressure (mmHg)	0.55	299

					Diastolic Blood Pressure (mmHg)	0.51	299
					Right CIMT (mm)	0.80	299
					Left CIMT (mm)	0.55	299
TET2	rs7678440	4	106398351	$PM_{2.5}$	Systolic Blood Pressure (mmHg)	0.68	302
					Diastolic Blood Pressure (mmHg)	0.97	302
					Right CIMT (mm)	0.93	302
					Left CIMT (mm)	0.87	302

[a] SNPs were modeled as ordinal variables (in which 0= minor allele, 1= heterozygote, and 2= major allele) and models were adjusted for admixture, sex, plate, *in utero* tobacco smoke and maternal education level. Interaction p-values were generated with Wald's test. The 2SD for the following 1st trimester pollutants $PM_{2.5}$, PM_{10}, NO_2, and O_3 are 14 µg/m^3, 32 µg/m^3, 21 ppb, and 44 ppb, respectively.

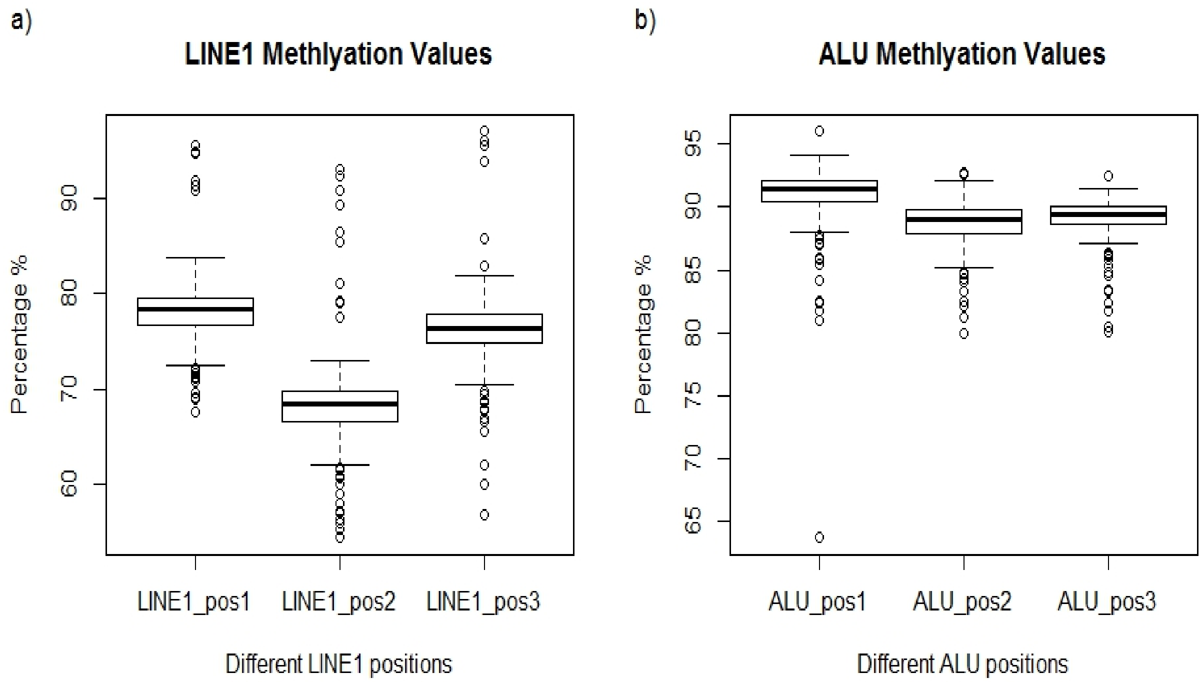

Figure S1. Boxplots of percent methylation for each assayed CpG in a) LINE1 (N=392) and b) AluYb8 (N=181). Boxes extend from the 25th to the 75th percentile, horizontal bars represent the median, whiskers extend 1.5 times the length of the interquartile range (IQR) above and below the 75th and 25th percentiles, respectively, and outliers beyond whiskers are represented as points.

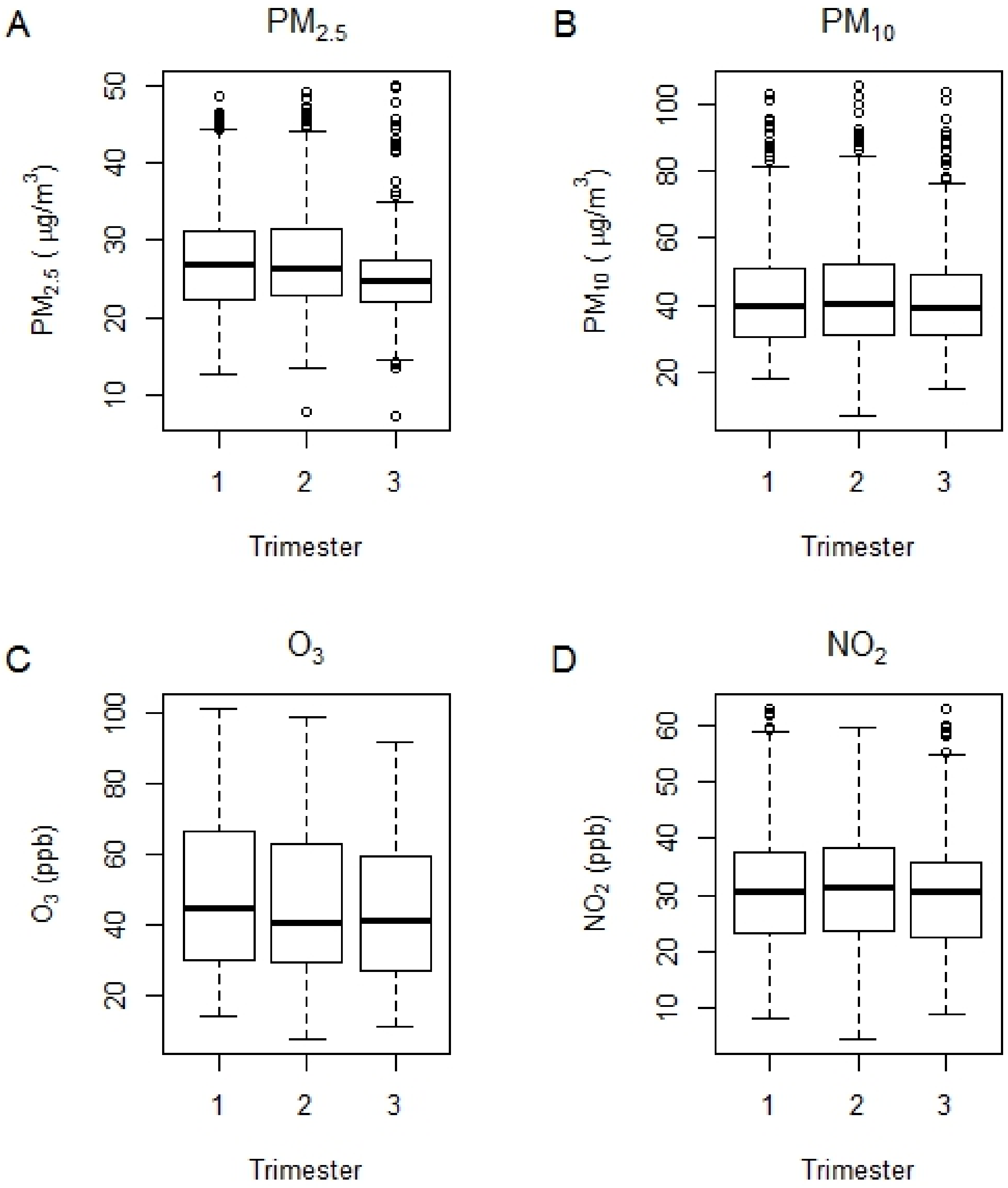

Figure S2. Distributions of ambient air pollutants by trimester (N=392). Boxes extend from the 25th to the 75th percentile, horizontal bars represent the median, whiskers extend 1.5 times the length of the interquartile range (IQR) above and below the 75th and 25th percentiles, respectively, and outliers are represented as points.

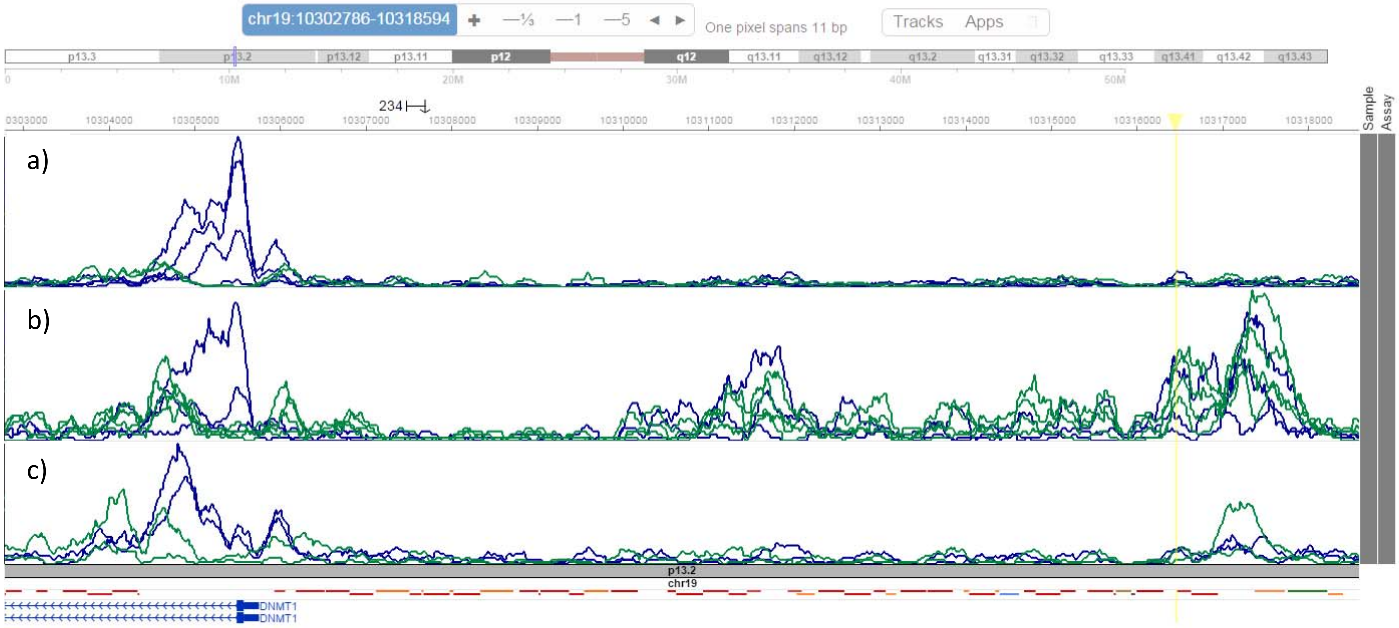

Figure S3. Illustration of rs16999714 (yellow bar) upstream of the DNMT1 transcription start site in a putative enhancer that appears a) inactive in H1 BMP4 derived mesendoderm cells, b) active in H1 BMP4 derived trophoblast cells, and c) adjacent to a poised enhancer in H1 derived mesenchymal stem cells. Blue is H3K27ac and green is H3K4me1. The figure was produced using the Wash U Epigenome Browser.[1]

References

1. Zhou X, Maricque B, Xie M, Li D, Sundaram V, Martin EA, Koebbe BC, Nielsen C, Hirst M, Farnham P, Kuhn RM, Zhu J, Smirnov I, Kent WJ, Haussler D, Madden PA, Costello JF and Wang T. The Human Epigenome Browser at Washington University. *Nat Methods*. 2011;8:989-990.
